# Dissemination of Internal Ribosomal Entry Sites (IRES) Between Viruses by Horizontal Gene Transfer

**DOI:** 10.3390/v12060612

**Published:** 2020-06-04

**Authors:** Yani Arhab, Alexander G. Bulakhov, Tatyana V. Pestova, Christopher U.T. Hellen

**Affiliations:** Department of Cell Biology, SUNY Downstate Health Sciences University, Brooklyn, NY 11203, USA; yani.arhab@downstate.edu (Y.A.); alexander.bulakhov@downstate.edu (A.G.B.); tatyana.pestova@downstate.edu (T.V.P.)

**Keywords:** IRES, *Flavivirus*, Horizontal gene transfer, *Hepacivirus*, *Pegivirus*, *Pestivirus*, Picornavirus, recombination, translation

## Abstract

Members of *Picornaviridae* and of the *Hepacivirus*, *Pegivirus* and *Pestivirus* genera of *Flaviviridae* all contain an internal ribosomal entry site (IRES) in the 5′-untranslated region (5′UTR) of their genomes. Each class of IRES has a conserved structure and promotes 5′-end-independent initiation of translation by a different mechanism. Picornavirus 5′UTRs, including the IRES, evolve independently of other parts of the genome and can move between genomes, most commonly by intratypic recombination. We review accumulating evidence that IRESs are genetic entities that can also move between members of different genera and even between families. Type IV IRESs, first identified in the *Hepacivirus* genus, have subsequently been identified in over 25 genera of *Picornaviridae*, juxtaposed against diverse coding sequences. In several genera, members have either type IV IRES or an IRES of type I, II or III. Similarly, in the genus *Pegivirus*, members contain either a type IV IRES or an unrelated type; both classes of IRES also occur in members of the genus *Hepacivirus*. IRESs utilize different mechanisms, have different factor requirements and contain determinants of viral growth, pathogenesis and cell type specificity. Their dissemination between viruses by horizontal gene transfer has unexpectedly emerged as an important facet of viral evolution.

## 1. Recombination as a Motive Force in Viral Evolution

Recombination, defined as the exchange of genetic elements between parental genomes to generate novel chimeras, occurs in divers positive-sense RNA viruses [1]. It can involve replicative or non-replicative mechanisms [2,3] and potentially serves functions that include the purging of deleterious mutations and the creation of advantageous novel genetic combinations to evade host immunity, gain resistance to antiviral agents, alter virulence and expand the host range [1,4,5]. Molecular epidemiological studies, for example, of circulating enteroviruses, have shown that recombination is a frequent occurrence in picornaviruses (e.g., [6,7,8]), and analysis of viral metagenomic data has emphasized the importance of recombination in the acquisition of novel sequences and, consequently, in the evolution of viruses [9,10]. In contrast to nucleotide substitution, which only allows gradual searching through evolutionary fitness space, recombination can lead to large shifts that can create beneficial genetic diversity but may also disrupt favorable combinations of co-adapted alleles [1,11]. This process of movement of genetic information between genomes is known as horizontal gene transfer, and it contrasts with the ‘vertical’ replicative transmission of genetic information from parents to offspring.

Studies of gene acquisition and dissemination by recombination have focused on protein-coding regions (e.g., [9,12,13,14]. For example, recombination between structural genes has influenced the host range expansion of coronaviruses by altering receptor usage [15] and the insertion of a cysteine protease from porcine torovirus (order *Nidovirales*) into the polyprotein encoded by enterovirus G (order *Picornavirales*) potentially antagonizes innate immune responses [16].

Less attention has been paid to the acquisition by RNA virus genomes of noncoding elements by horizontal gene transfer, even though such entities are heritable, genetically stable, and have specific functions in key aspects of the viral life cycle that include translation, replication and encapsidation [17,18,19]. Noncoding elements can determine viral host range, cell type specificity and pathogenesis (e.g., [20,21,22,23]. For example, internal ribosomal entry sites (IRESs) are large, structurally complex RNAs that mediate cap- and 5′-end-independent initiation of translation [24,25] and that confer selective advantages on viral mRNAs, particularly during infection. Here, we will discuss IRESs as an example of a class of noncoding RNA element that can move between the genomes of positive-strand RNA viruses by recombination.

## 2. Viral Infection and Translational Control

With the exception of ‘giant viruses’, viruses do not encode translation factors, and are thus wholly reliant on the host translational apparatus for synthesis of viral proteins [25]. Viral infection leads to competition between viral and cellular mRNAs for access to the protein synthetic machinery, and viruses and their hosts have consequently developed measures and counter measures to promote translation of their own mRNAs.

Translation consists of initiation, elongation, termination and ribosome recycling phases [24]. The canonical initiation process begins with recycling of post-termination ribosomes to generate a pool of free 40S and 60S ribosomal subunits [26]. Aminoacylated initiator tRNA, eukaryotic initiation factor 2 (eIF2) and GTP form a ternary complex (eIF2-TC), which is recruited with eIF1, eIF1A, eIF3 and eIF5 to a 40S subunit, yielding a 43S pre-initiation complex. Eukaryotic mRNAs are post-transcriptionally modified by the addition of a triphosphate-linked 7-methylguanosine (m^7^GTP) cap at their 5′-termini, and one or two cap-adjacent nucleotides may also be 2′-*O*-methylated. This ‘cap’ is bound by eIF4F, a heterotrimer comprising the cap-binding protein eIF4E, eIF4G and eIF4A, a RNA helicase. eIF4F recruits the 43S complex to the 5′-end of the mRNA [27], and it then scans in a 5′-3′ direction along the mRNA until it encounters an AUG initiation codon. Establishment of base pairing between the initiation codon and the anticodon of initiator tRNA in the resulting 48S complex triggers eIF5-induced hydrolysis of eIF2-bound GTP, followed by eIF5B-mediated reorientation of tRNA, joining of a 60S subunit and release of initiation factors to form a 80S ribosome that is primed to begin elongation. The elongation process consists of the coordinated decoding of the open reading frame (ORF) and synthesis of the encoded polypeptide.

Cells counter viral infection by activation of innate immune responses, which include synthesis of ‘Interferon-induced protein with tetratricopeptide repeats’ (IFIT) proteins that sequester the 5′-terminal cap of viral mRNAs that lack 2′-*O*-methylated cap-proximal nucleotides (e.g., [28]), and phosphorylation of the eIF2a subunit by protein kinase R [25]. Viruses may suppress these responses, and in addition, may inhibit cellular translation by abrogating or altering the activity of initiation factors; for example, as a result of their cleavage by virus-encoded proteases (e.g., [29]) or by activation of translational repressors [30]. Viral mRNAs continue to gain access to the translation apparatus in these circumstances by using alternative mechanisms of initiation that require only a subset of initiation factors. Many positive-sense RNA viruses that induce changes in the cellular translation apparatus contain an IRES that promotes cap- and 5′-end-independent initiation of translation. IRESs function by interacting in a specific, non-canonical manner with one or more components of the canonical translation apparatus, leading to recruitment of ribosomes or ribosomal pre-initiation complexes to an internal location on the mRNA [25]. The presence of an IRES in viral mRNAs enables them to escape IFIT-mediated impairment of initiation, and to function despite virus-induced inactivation of one or more initiation factors, and in some instances, despite phosphorylation of eIF2.

## 3. Classification of Viral IRESs

IRESs occur in a broad range of positive-sense RNA viral genomes, including those of members of *Picornaviridae*, *Flaviviridae*, *Dicistroviridae* and *Iflaviridae* families, as well as in several retroviruses [19,25,31]. IRESs are classified into a few major groups, each of which has a distinct structural core that is maintained by sequence co-variation [32], and each of which uses a distinct mechanism to initiate translation.

**Figure 1:**
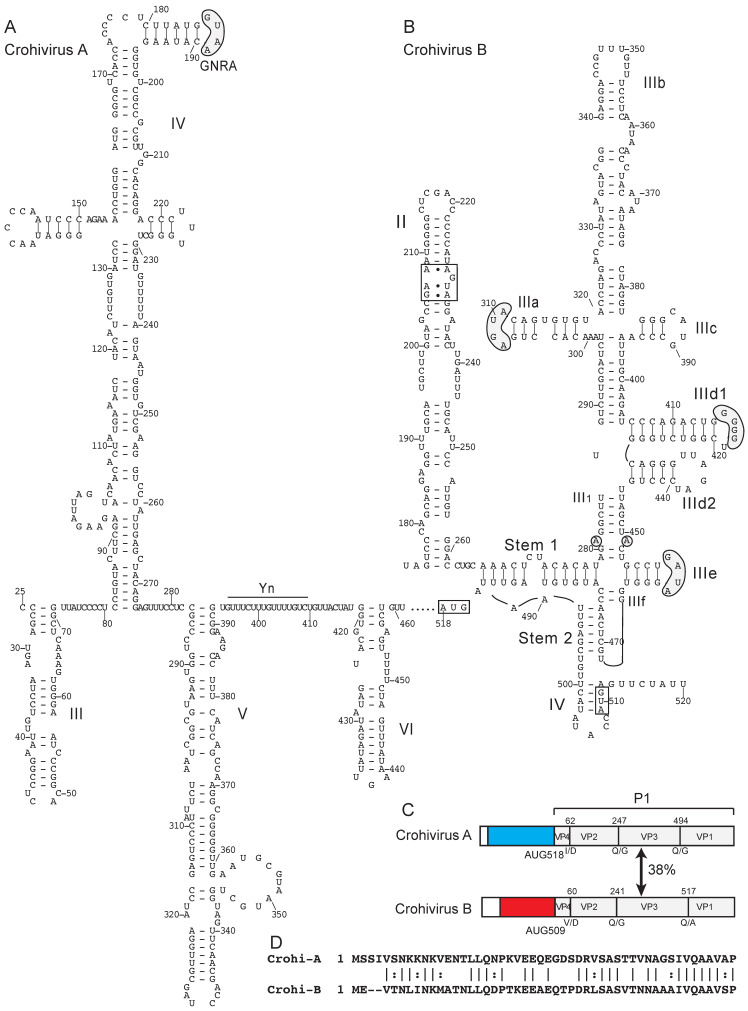
Two different classes of IRES in the genomes of members of the genus *Crohivirus* of *Picornaviridae*. (**A**) Model of the structure of the type I IRES of crohivirus A strain ZM54 (NCBI ref. seq. NC_025474.1) and (**B**) Model of the structure of the type IV IRES of crohivirus B clone Bat/CAM/CroV-P25/2013 (NCBI ref. seq. NC_033819.1). Nucleotides are numbered and the initiation codon is boxed. Domains are labeled (A) III – VI and (B) II – IV. The IRES structures have been annotated (A) to show the GNRA tetraloop at the apex of domain IV and the pyrimidine (Yn) tract of the Yn-Xm-AUG motif and (B) to show the loop-E motif in domain II and to highlight sequence motifs in domain III that are conserved in other type IV IRESs, such as the apical loops in subdomains IIIa, IIId and IIIe, and the unpaired purine residues in helix III_1_ (grey shading). The tertiary base-pairing interaction between one of these unpaired purine residues in helix III_1_ and a flipped-out pyrimidine residue in the loop of subdomain IIIe is indicated by a blue line. (**C**) Schematic representations of the 5′UTRs and adjacent coding region for the P1 capsid protein precursor, annotated to show noncoding regions that constitute the type I IRES in crohivirus A (light blue) and the type IV IRES in crohivirus B (red), the N-termini of the structural proteins VP4, VP2, VP3 and VP1 and the protease cleavage sites between them, and the amino acid sequence identity between the two P1 polyproteins. (**D**) Sequence alignment of the N-terminal regions of the VP4 proteins of crohivirus A and crohivirus B. Identical and related residues are indicated by lines and colons, respectively.

**Figure 2:**
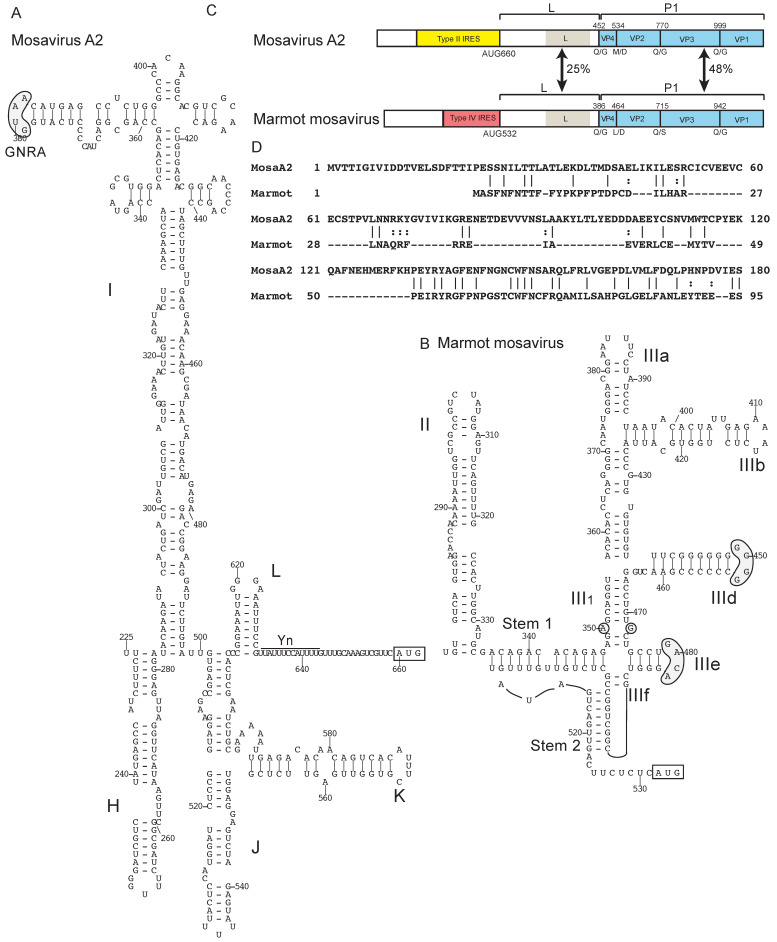
Two different classes of IRES in the genus *Mosavirus* of *Picornaviridae*. (**A**) Model of the structure of the type II IRES of mosavirus A2 strain SZAL6-MoV/2011/HUN (NCBI ref. seq. NC_023987.1) and (**B**) model of the structure of the type IV IRES of mosavirus B marmot mosavirus strain HT8 (Genbank: KY855435). Nucleotides are numbered and the initiation codon is boxed. Domains are labeled (A) H – L and (B) II – III. The IRES structures have been annotated (A) to show the GNRA tetraloop at the apex of domain I and the Yn-Xm-AUG motif and (B) to highlight sequence motifs in domain III that are conserved in other type IV IRESs (grey shading). (**C**) Schematic representations of the 5′UTRs and adjacent coding region for the Leader (L) protein and the P1 capsid protein precursor (light blue), annotated to show noncoding regions that constitute the type II IRES in mosavirus A (yellow) and the type IV IRES in mosavirus B (rose), the L protein and the most conserved elements in it, the N-termini of the structural proteins VP4, VP2, VP3 and VP1 and the protease cleavage sites between them, and the amino acid sequence identity between the two L proteins and between the P1 polyproteins. (**D**) Sequence alignment of the N-terminal regions of the L proteins of mosavirus A2 and marmot mosavirus. Identical and related residues are indicated by lines and colons, respectively.

Type I and Type II IRESs are epitomized by poliovirus (PV) (of the genus *Enterovirus* of *Picornaviridae*) and encephalomyocarditis virus (EMCV) (of the genus *Cardiovirus* of *Picornaviridae*), respectively [25]. These ~450 nt long IRESs have distinct conserved structures (Figure 1A and Figure 2A) but share some functionally important sequence motifs, such as an apical tetraloop in the largest domain and a Yn-Xm-AUG motif at the 3′-border of the IRES in which a pyrimidine-rich tract (Yn; n = 8–10 nt) is separated from an AUG triplet by a spacer (Xm; m = 18–20 nt). Both classes of IRES can promote initiation at this AUG codon as well as at AUG codons that can be as much as ~150 nt downstream and that may or may not be in-frame [33,34,35]. Viruses that contain these types of IRES commonly encode proteases that cleave eIF4G, splitting the N-terminal domain that binds eIF4E from the domains that bind eIF4A and eIF3 (e.g., [29]). Both classes of IRES function in the absence of eF4E and this domain of eIF4G [36,37,38], and instead, depend on a direct, non-canonical interaction with eIF4G’s central domain that recruits eIF4A to the IRES [39,40,41]. Binding of these factors to the IRES and its subsequent restructuring are prerequisites for recruitment of the 43S complex [36,37]. In addition to canonical eIFs, the activity of type I and type II IRESs involves IRES *trans*-acting factors (ITAFs), which are cellular RNA-binding proteins that are thought to assist the IRES in attaining an active conformation (e.g., [42]). The dependence on ITAFs and the identity of the ITAF(s) required for activity is IRES specific. The pyrimidine tract-binding protein PTB is required by types I and II [43,44,45], whereas the poly(C)-binding protein 2, upstream of *N-ras* and glycl-tRNA synthetase have only been implicated in type I IRES function and ITAF_45_/Ebp1 enhances the activity of the type II IRES of foot-and-mouth disease virus (FMDV) (genus *Aphthovirus*) [38,46,47,48,49]. Differences in expression levels of critical ITAFs are thought to underlie the cell type specificity of both classes of IRES.

Type III IRESs were first identified in the 734 nt-long 5′UTR of hepatitis A virus (genus *Hepatovirus* of *Picornaviridae*) [50]. This 5′UTR comprises six domains: domains IV and V form the essential core of the IRES and are located immediately upstream of a Yn-Xm-AUG motif, in which the AUG triplet is the initiation codon for the viral polyprotein [51]. Initiation activity is weak, is augmented by ITAFs, and depends on binding of eIF4G to the IRES as a constituent of intact eIF4F, but the full set of factors required for initiation on this IRES has not been established [52,53]. The HAV IRES domain that is recognized by eIF4G [54] topologically resembles the J-K domain of type II IRESs to which eIF4G binds [41].

Type IV IRESs were initially identified in hepatitis C virus (HCV) of the *Hepacivirus* genus, and in bovine viral diarrhea virus (BVDV) and classical swine fever virus (CSFV) of the *Pestivirus* genus of *Flaviviridae* [55,56,57] and as described below, variant forms have subsequently been identified in numerous genera of *Picornaviridae*. In members of *Hepacivirus* and *Pestivirus* and in some members of *Picornaviridae* (e.g., Figure 1B), these ~300 nt-long IRESs contain two major domains (II and III), and in some instances, the minor domain IV, a hairpin structure that includes the initiation codon [58]. The liver-specific microRNA-122 (miR-122), which is a determinant of the hepatotropism of HCV, binds to two sites upstream of the HCV IRES and promotes RNA stability, translation and replication [59]. Domain III consists of a series of stemloops (IIIa – IIIf) that form three- or four-way junctions. It is strongly conserved, particularly at the apex of IIId (which contains a characteristic exposed GGG motif) and in the basal region, which contains a pseudoknot and a stemloop (IIIe) with a universally conserved apical GA[U/C]A tetraloop that base pairs with a bulged nucleotide in the adjacent helix III_1_ [58,60]. The apical region of domain III contains subdomains IIIa and IIIc that interact with the 40S subunit, for example via the conserved apical ‘AGUA’ loop in IIIa [61,62]; this apical region also binds to eIF3 [63]. Domain II is a flexible 60–75 nt-long hairpin that in many instances contains a loop-E motif [58]. Its role is to facilitate steps in initiation leading up to subunit joining [64,65]. Initiation on these IRESs involves direct binding of eIF3, and multipoint interaction of the 40S subunit with stemloops IIIa, IIIc, IIId, IIIe and the pseudoknot [61,62,63,66,67,68]. Base pairing of the apical GGG motif in IIId with expansion segment 7 (ES7) of 18S ribosomal RNA (rRNA) is critical for the affinity of the IRES-40S interaction [68] and induces a structural rearrangement in the resulting complex that likely primes it for binding of initiator tRNA and start codon recognition [69,70]. These interactions with the 40S subunit enable type IV IRESs to initiate translation without the involvement of eIF4F. Moreover, the IRES-mediated positioning of the initiation codon in the ribosomal P site without prior scanning enables the IRES to promote initiation both by an eIF2-dependent mechanism [66,71] and by an eIF2-independent mechanism in which eIF5B stabilizes binding of initiator tRNA and orients it on the 40S subunit prior to subunit joining [65,72].

Type V IRESs have been identified in *Kobuvirus*, *Oscivirus*, *Passerivirus* and *Salivirus* genera of *Picornaviridae* [73,74,75]. Their domain organization resembles that of type I and type II IRESs, with a class-specific domain I at the 3′-border followed by a large cruciform domain J (with an apical GNRA tetraloop) that resembles domain IV of type I IRESs, the eIF4G-binding domain K that contains motifs that are characteristic of type II IRESs, a Yn-Xm-AUG motif at the 3′ border and a hairpin domain that sequesters this AUG triplet, which is the initiation codon for the viral polyprotein. The apparent hybrid nature of type V IRESs suggests that they may be chimeras formed by recombination between type I and type II IRES.

The genomes of members of *Dicistroviridae* and related viruses are dicistronic and contain IRESs in the 5′UTR and in the intergenic region (IGR) [76]. The 5′UTR IRESs have not been classified but are structurally diverse and utilize distinct mechanisms for initiation [77,78]. IGR IRESs that have been characterized to date fall into two structural groups, exemplified by cricket paralysis virus (CrPV) and Taura syndrome virus (TSV). Both are ~200 nt long and consist of one domain that comprises two nested pseudoknots and a second domain that consists of a single pseudoknot (in CrPV) or of a pseudoknot with a protruding hairpin (in TSV). This domain mimics the anticodon stemloop of tRNA base paired to an mRNA codon. These IRESs bind directly to the 40S subunit and recruit a 60S subunit to form an active initiation complex without the involvement of initiation factors, initiator tRNA or an initiation codon [79,80,81]. The bound IRES occupies the ribosomal A site and must be translocated to the P site by elongation factor 2 (eEF2) before an aminoacyl-tRNA/eIF1A-GTP complex can bind to the A site to allow elongation to occur [82]. IGR IRES function is independent of eIF2 and is upregulated by phosphorylation of eIF2a, likely because of the greater availability of ribosomes when translation of cellular mRNA is impaired [79]. This mechanism therefore exploits the innate immune response to promote viral replication.

A few groups of viral IRESs, each with related sequences and conserved structures, have not yet been incorporated into this classification scheme. For example, the 5′UTRs of pegivirus A and pegivirus C, members of the *Pegivirus* genus of *Flaviviridae*, contain functional IRESs [83]. The ~600 nt-long 5′UTRs both have a 3′-terminal Yn-Xm-AUG motif, but otherwise appear to be unrelated to other classes of IRES. The mechanism by which they function has not been determined.

## 4. Intertypic and Intratypic Recombination in the *Picornaviridae*

Phylogenetic analysis of prototype and field strains of human enteroviruses and rhinoviruses revealed that recombination in them is highly prevalent and identified the junction of the 5′UTR and the P1 capsid protein region as a hot-spot for recombination (e.g., [7,84,85,86,87,88]. The 5′UTRs of all members of the *Enterovirus* genus of *Picornaviridae*, including human and non-human enteroviruses and human rhinoviruses, contain type I IRESs. The finding that the 5′UTRs of human enteroviruses segregate into two clades whereas the coding region and 3′UTR form four distinct genetic groups that are now designated as the species human enterovirus (HEV)-A to HEV–D, and the identification of the 5′UTR/VP4 junction as a common locus for recombination, suggested that the 5′UTR is a distinct genetic unit that evolves independently of other elements of the viral genome in which it resides [84,89]. Studies of recombinant enterovirus replicons and viruses established that the enterovirus 5′UTR is modular, consisting of the IRES and a functionally independent 5′-terminal cloverleaf element involved in genome replication [90] and that intertypic sequence exchanges in the 5′UTR are tolerated but may lead to general or cell type-specific growth defects and attenuation of virulence [90,91,92,93,94]. Here, we define intratypic recombination as occurring between different strains of the same species, and intertypic recombination as occurring between virus species within the same genus.

Intratypic recombination events involving exchange of the 5′UTR have been extensively documented in human and non-human enterovirus species (e.g., [85,86,95]). The PV IRES contains determinants of pathogenicity and cell type-specificity and mutations in the IRESs of live PV vaccine strains that attenuate their neurovirulence can revert by point mutation [20]. Numerous circulating vaccine-derived poliovirus (cVDPV) genomes contain a 5′UTR that has been acquired by intratypic recombination from a different strain of the same enterovirus species, and that therefore lacks attenuating mutations. Recombination therefore likely contributes critically to the emergence of cVDPV genomes that have been identified in outbreaks of poliomyelitis in regions of the world with suboptimal vaccination coverage (e.g., [96,97]).

Intertypic recombination occurs at a much lower frequency than intratypic recombination [98], and consistently, only a few instances of intertypic recombination leading to acquisition of a heterologous 5′UTR have been reported. They are associated with the emergence of novel human picornavirus species such as *Enterovirus (EV) 90*, *EV91* [99], *EV104* [100] and *EV109* [101], and novel animal picornaviruses such as ovine enterovirus-1, in which the coding sequence most closely matches porcine enterovirus 10, a member of the species *Porcine enterovirus B*, whereas the 5′UTR is closely related to those of bovine enteroviruses [102]. Intertypic recombination leading to the acquisition of IRES elements therefore influences the pathogenesis of enteroviruses and can promote their speciation. This is likely also the case for all other picornaviruses, since there is significant evidence for the intertypic exchange of type II IRESs between members of the genera *Aphthovirus* [103] and of *Parechovirus* [104,105], and for the intertypic exchange of type IV IRESs between members of the genus *Teschovirus* [103].

## 5. Experimental Exchange of IRESs between Members of Different Viral Genera and Families

Substitution of the EMCV IRES for the native IRES in PV and HAV [106,107] was tolerated without alteration of viral growth characteristics, whereas replacement of the poliovirus IRES by the HCV IRES and part of the adjacent coding region yielded a genetically stable virus with strongly reduced growth kinetics [108]. Viral IRESs can therefore function in the context of heterologous viral genomes.

However, type I and some type II IRESs have strong requirements for ITAFs that likely underlie cell type-specific differences in IRES activity. Consistently, some IRES exchanges are associated with significant impairment of viral translation in specific cell types and with attenuation of virulence. Replacement of the PV IRES by that of HRV-2 yielded a chimeric virus that grew better than the wild-type virus in human epithelial type 2 (HEp-2) cells, but its growth was almost abrogated in neuronal cells and it lacked neurovirulence in a mouse model and in *Cynomolgus* monkeys [92,109]. Determinants of tropism for cells of neuronal origin mapped to apical regions of IRES domains V and VI. Replacement of the 5′UTR of a virulent strain of Coxsackie virus B3 that causes severe pancreatic and heart disease in mice by the PV 5′UTR yielded a chimeric virus that had strongly attenuated growth in murine fetal heart fibroblasts and caused transient mild disease in mice even though its growth in HeLa cells was unaffected [93]. The Theiler’s murine encephalomyocarditis virus (TMEV) GDVII strain causes fatal encephalitis in mice following intercranial inoculation, but it was completely attenuated in mice following substitution of its IRES by that of the FMDV O_1_K strain, even though substitution had little or no effect on translation in vitro and in baby hamster kidney (BHK) 21 cells, and the chimeric viral genome was infectious and the plaque size of progeny was only slightly reduced [48]. Notably, initiation on these two type II IRESs has different factor requirements: they need the same set of canonical initiation factors but whereas the TMEV IRES requires PTB, the FMDV IRES requires PTB and ITAF_45_/Ebp1.

## 6. Horizontal Gene Transfer of Type IV IRESs in Picornaviruses

Type IV IRESs were discovered in the *Hepacivirus* and *Pestivirus* genera of *Flaviviridae*, and their subsequent identification in porcine teschovirus 1 of the genus *Teschovirus* of *Picornaviridae* [110] suggested that this class of IRES could be disseminated between unrelated viruses by horizontal gene transfer. Type IV IRESs have subsequently been identified in numerous other genera of *Picornaviridae*, including *Aalivirus*, *Anativirus*, *Aquamavirus*, *Avihepatovirus*, *Colbovirus*, *Felipivirus*, *Hepatovirus*, *Kobuvirus*, *Limnipivirus*, *Livupivirus*, *Megrivirus*, *Parechovirus*, *Pasivirus*, *Phacovirus*, *Sakobuvirus*, *Sapelovirus*, *Senecavirus* and *Tremovirus* [111,112,113,114,115,116,117,118,119,120,121,122,123,124,125,126,127,128,129,130,131,132]. Biochemical studies have confirmed that these IRESs promote initiation by the same eIF4F-independent mechanism as HCV and pestivirus IRESs [110,133,134,135]. The presence of type IV IRESs in so many viruses from different families and genera, juxtaposed against unrelated coding sequences constitutes strong support for the hypothesis that this class of IRES can be acquired and disseminated by horizontal gene transfer.

Although some structural heterogeneity is apparent in type IV IRESs from members of *Flaviviridae*, such as the presence of an additional subdomain (IIId2) in pestivirus IRESs and of two additional subdomains (IIb and IIc) in hepacivirus B IRESs, all of which contribute to function [136,137], the structural diversity of type IV IRESs in picornaviruses is nevertheless remarkable. These IRESs all contain an essential, highly conserved core comprising the pseudoknot (PK) and subdomains IIId, IIIe and IIIf. The apical GGG motif in domain IIId is invariant, consistent with its critical function in engaging with the ribosomal 40S subunit via a base-pairing interaction with the UCCC loop of ES7 of 18S rRNA [63], and its functional importance in picornavirus type IV IRESs [133,134,138]. The apical UCCC loop occurs in ES7 of 18S rRNA and is conserved in a wide range of vertebrate animals, consistent with the exploitation of type IV IRESs by viruses that infect this diverse range of hosts. The apical tetraloop in domain IIIe of picornavirus type IV IRESs contains a flipped-out pyrimidine residue in the third position that engages in a co-variant base-pairing interaction with a bulged nucleotide in the adjacent helix III_1_ [60]. As in hepaciviruses, this interaction is thought to stabilize the orientation of helices in the pseudoknot, which functions in ribosomal binding and in promoting correct positioning of the coding region in the mRNA-binding cleft of the 40S subunit [139]. The structure of the pseudoknot in these IRESs is maintained by sequence covariation, and consistently, second-site substitutions restore function in IRESs that had lost activity due to destabilizing substitutions [111].

In contrast to the conserved core, peripheral elements of picornavirus type IV IRESs are highly variable. Domain II may be shorter or longer than in the HCV IRES, may or may not contain a loop-E motif, and in the *Teschovirus* and *Tremovirus* genera, contains a small branching hairpin. The apical ‘IIIabc’ region of domain III may be severely truncated, for example in members of *Kobuvirus*, *Kunsagivirus*, *Limnipivirus* and *Rafivirus* genera, may lack one (*Kunsagivirus*, *Limnipivirus*, *Sapelovirus* and *Teschovirus* genera) or two subdomains (*Aalivirus* genus); sub-domains IIIa and IIIc may also be disposed in a staggered configuration rather than forming a four-way junction and IIIb may be greatly elongated (e.g., in the *Megrivirus* genus). Many of these IRESs do not have a propensity to form domain IV.

The advent of metagenomic analysis of viral sequences has led to a remarkable increase in the number of members of *Picornaviridae*, notably from host species that have historically been under-sampled (e.g., [140,141]). We analyzed novel picornavirus sequences to identify additional type IV IRESs, to expand knowledge of the prevalence of IRES dissemination by horizontal gene transfer and of the structural repertoire of type IV IRESs, and potentially, to gain further insights into the mechanisms that gave rise to variant IRESs. Numerous novel genera of *Picornaviridae* have been recognized in recent years, and we will begin with a consideration of genera in which the IRES has not yet been characterized.

## 7. Unrelated IRESs Within Individual Picornavirus Genera: Evidence for Horizontal Gene Transfer? 

The presence of more than one type of IRES in otherwise related genomes of viruses from the same genus is a strong indication that heterologous IRESs have been acquired by recombination. For example, aichivirus A and aichivirus B in the genus *Kobuvirus* contain type V IRESs [74], whereas aichivirus C in this genus contains a type IV IRES [117]. Similarly, most members of the *Parechovirus* genus contain type II IRESs, but type IV IRESs have been identified in parechovirus D (ferret parechovirus) (GenBank: NC_034453.1) [142] and in Manhattan parechovirus (GenBank: KJ950935) [130]. Parechovirus sp. QAPp32 (Genbank: MK348056), isolated from the bat *Pipistrellus pipistrellus*, also has a type IV IRES that shares 80% sequence identity with parechovirus D, and encodes a polyprotein that is ~40% identical to those of parechovirus B (Ljungan virus) and parechovirus C (Sebokele virus). This virus therefore likely also acquired a type IV IRES by horizontal gene transfer. A complementary observation is that members of the genus *Hunnivirus* encode polyproteins that are related to those encoded by porcine teschoviruses (genus *Teschovirus*), but whereas teschoviruses contain type IV IRESs, these hunnivirus genomes contain Parechovirus-like type II IRESs [143]. 

Crohivirus A of the genus *Crohivirus* has a type I IRES (Figure 1A) that shares extensive homology with the IRES of the unclassified Bat picornavirus 3 (NCBI ref. seq.: NC_015934) [120] (71% identity over ~350 nt) and with IRESs from members of the genus *Enterovirus* (70%–80% identity in domain V). By contrast, crohivirus B [144] has an IRES that is a homologue of the parechovirus D IRES (56% sequence identity) and of type IV IRESs of pestiviruses (for example, 52% sequence identity with the CSFV IRES (GenBank: LT593748.1)). Thus, domain III of the crohivirus B IRES contains an additional hairpin (subdomain IIId2) in addition to the complete complement of characteristic subdomains and motifs, and the less conserved domain II nevertheless contains a loop-E motif (Figure 1B). Sequence identity between the crohivirus A and crohivirus B polyproteins extends almost to their N-terminus, suggesting that recombination likely occurred close to the junction of the 5′UTR and the VP4 coding sequence in ancestral strains of these viruses (Figure 1C,D).

Mosavirus A2 of the genus *Mosavirus* of *Picornaviridae*, which was isolated from *Coracias garrulus*, an afro-palearctic migratory bird, has a type II IRES with limited sequence homology to type II IRESs from members of *Cosavirus*, *Mischivirus* and *Parechovirus* (Figure 2A; [145]). On the other hand, mosavirus B (marmot mosavirus) [146] has a type IV IRES (Figure 2B) that is related to those of the genus *Teschovirus*: domain II is relatively short and lacks a loop-E motif, there is no subdomain IIIc, the apical loop of subdomain IIId contains only two contiguous G residues and the apical loop of subdomain IIIe has the sequence GACA but retains the ability to interact with the adjacent helix III_1_ by base pairing. The lack of homology between the N-terminal regions of the Leader proteins at the N-terminus of the viral polyprotein encoded by these viruses (Figure 2C,D) suggests that the recombination breakpoint in ancestors of these viruses maps to this region.

**Figure 3:**
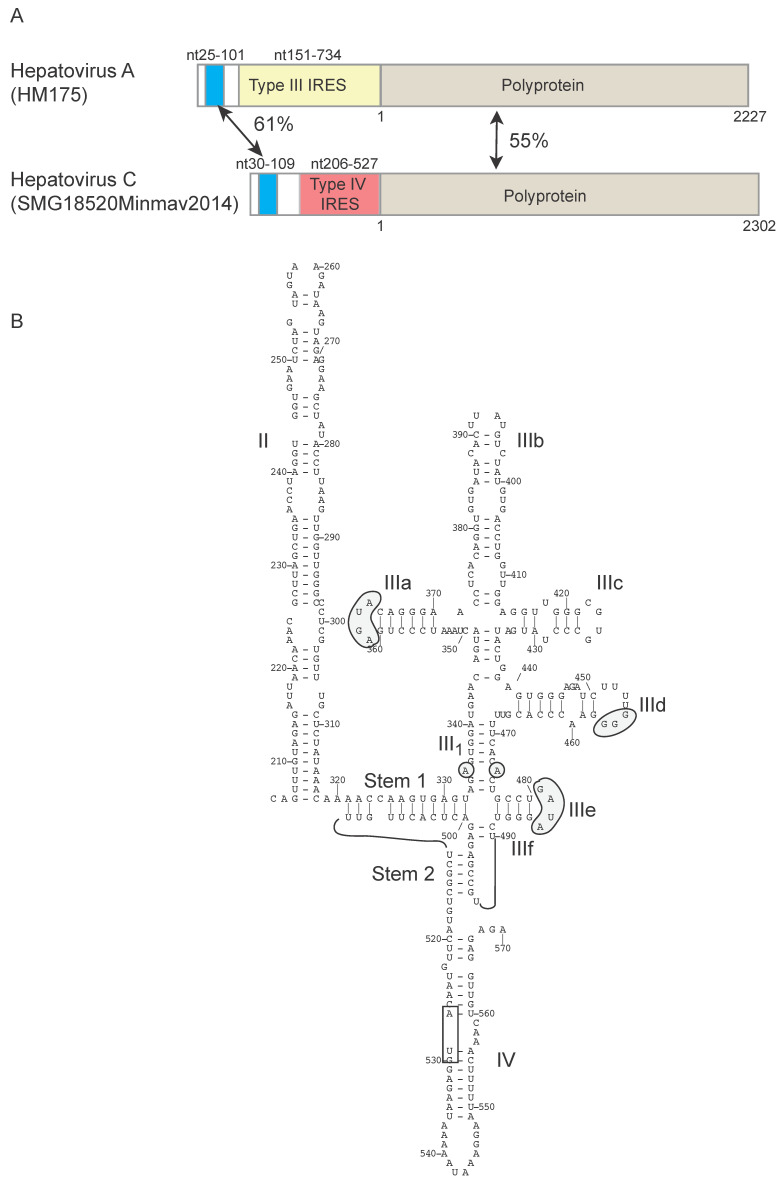
A type IV IRES in the genome of a member of the genus *Hepatovirus*. (**A**) Schematic representations of the 5′UTRs and adjacent coding region of hepatovirus A isolate HM175 (NCBI ref. seq. NC_001489.1) [147] and of hepatovirus C isolate SMG18520Minmav2014 (NCBI ref. seq. NC_038313.1) [131]. The 5′UTRs are divided to show a homologous element near the 5′-end (shaded light blue), the type III IRES in hepatovirus A and the type IV IRES in hepatovirus C. The nucleotides forming these noncoding elements and the N- and C-terminal amino acid residues of the polyprotein are numbered, and the nucleotide or amino acid sequence identity between them is indicated, as appropriate. (**B**) The model of the secondary and tertiary structure of this hepatovirus C IRES was derived as described for related IRESs [114,130]. Domains are labeled II - IV, subdomains are labeled IIIa, IIIb etc, Stem 1 and Stem 2 are elements of the pseudoknot, and helix III_1_ is the basal element of domain III. The initiation codon for the viral polyprotein (AUG_528_) is indicated by black box. Sequence motifs in domain III that are characteristic of type IV IRESs are indicated by grey shading.

Analysis of the 5′UTRs of members of the genus *Hepatovirus* derived from a global collection of 209 species of small mammals identified two that contained type IV IRESs [131]. For example, the 5′UTR of hepatovirus C from a bat from Madagascar contains a canonical type IV IRES flanked on both sides by hepatovirus A-like sequences. The upstream 5′-terminal region shares 61% sequence identity with a similarly located segment in the 5′UTR of hepatovirus A, and the downstream ORF encodes a polyprotein that shares ~55% identity with the hepatovirus A polyprotein, with homology extending to within a few amino acid residues of the N-terminus (Figure 3). These observations suggest that the evolution of this hepatovirus C isolate may at some stage have involved ‘capture’ of a type IV IRES by a hepatovirus A-like genome, leading to replacement of the type III IRES by a double recombination event.

**Figure 4:**
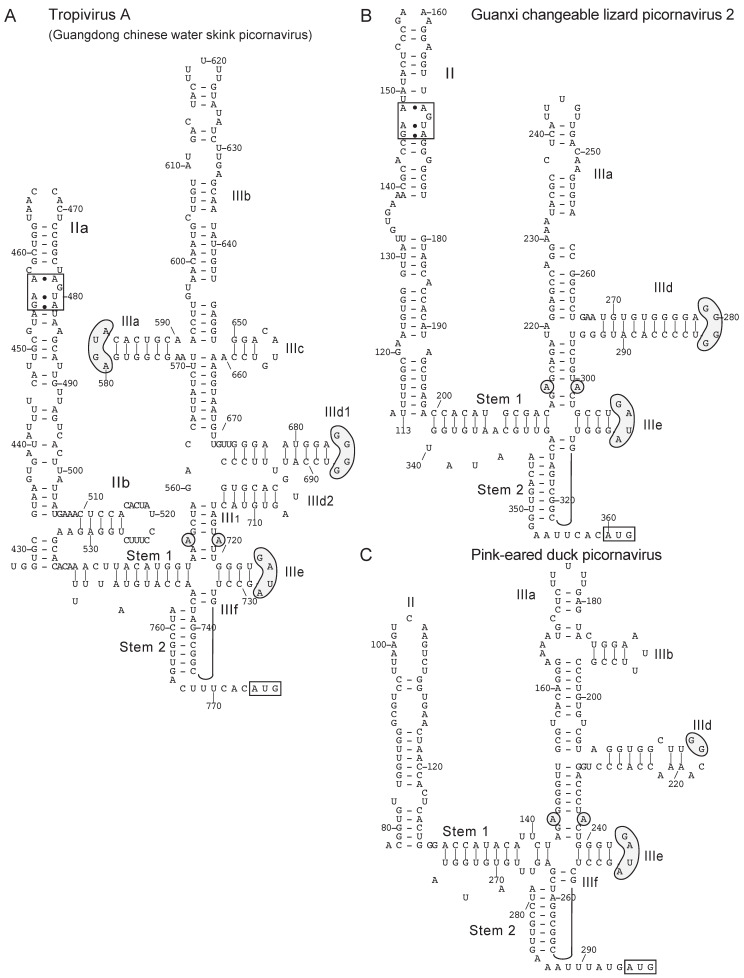
Structurally diverse type IV IRESs in novel picornavirus genomes. (**A**) Model of the Tropivirus A (Guangdong Chinese water skink picornavirus) IRES (GenBank: MG600091) [141], (**B**) Model of the Guanxi changeable lizard picornavirus 2 IRES (GenBank: MG600105) [141] and (**C**) Model of the Pink-eared duck picornavirus IRES (GenBank: MK204421) [148], derived as described for related IRESs [114,130]. Nucleotides are numbered at intervals, the initiation codon for the viral polyprotein is boxed, and domains and helices are labeled using the accepted nomenclature [114]. The loop-E motif in domain II and conserved sequence motifs in subdomains IIIa, IIId and IIIe and the unpaired purine residues in helix III_1_ are indicated by gray shading.

Tropivirus A (Guangdong Chinese water skink picornavirus) [141] of the genus *Tropivirus* has a type IV IRES with all conventional subdomains and motifs, and additional branching stemloops in domain II (IIb) and domain III (IIId2). Homology is greatest with the rafivirus IRES [130], which also has a IIId2 subdomain, and homology extends from subdomain IIIc to the initiation codon (Figure 4A). However, the polyprotein encoded by tropivirus A is more closely related to polyproteins encoded by members of the genus *Kobuvirus* than of the genus *Rafivirus*, suggesting that this virus may also have a chimeric genome with the IRES and open reading frame deriving from different ancestral genomes.

Taken together, six different genera of *Picornaviridae* (*Crohivirus*, *Hunnivirus*, *Hepatovirus*, *Kobuvirus*, *Mosavirus* and *Parechovirus*) contain members that encode homologous polyproteins but have different types of IRES. Each genus has a member that contain a type IV IRES in addition to members that contain a type I IRES (*Crohivirus*), type II IRESs (*Hunnivirus*, *Mosavirus* and *Parechovirus*), type III IRESs (*Hepatovirus*) or type V IRESs (*Kobuvirus*). Tropivirus A of the genus *Tropivirus* may also have a chimeric genome in which the IRES and the coding region have different genetic origins. These observations provide further evidence for the prevalence of horizontal gene transfer of IRESs in members of *Picornaviridae*.

## 8. Novel Picornavirus Type IV IRESs with a Conserved Sapelovirus-Like Core

Simian and porcine sapelovirus type IV IRESs diverge from canonical HCV-like type IV IRESs in that domain II may lack a loop-E motif, subdomain IIIc is absent and subdomain IIIb is greatly elongated [114]. Many other members of the genus Sapelovirus, as well as several unassigned picornaviruses, contain IRESs with high sequence homology to, e.g., porcine sapelovirus IRESs, particularly in the PK-IIId-IIIe-IIIf core, but have a shorter subdomain IIIb [130]. This conserved core of domain III is closely related to analogous sequence in type IV IRESs from members of the genera *Felipivirus* and *Teschovirus*. Here, we describe additional type IV IRESs with a sapelovirus-like core in members of several genera of Picornaviridae and in numerous unassigned viral sequences.

**Figure 5:**
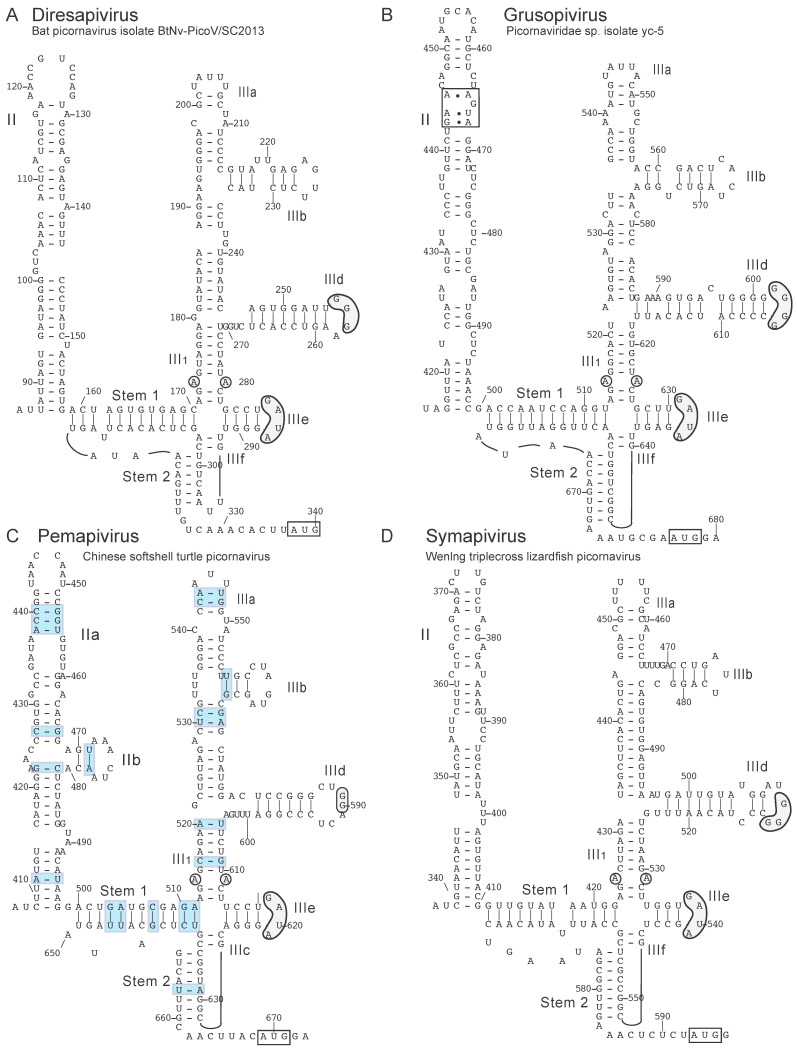
Type IV IRESs in members of the genera *Diresapivirus*, *Grusopivirus*, *Pemapivirus* and *Synapivirus*. (**A**) Model of the structure of the diresapivirus B1 IRES (GenBank: KJ641697.1) (genus *Diresapivirus*), (**B**) Model of the structure of the grusopivirus A1 IRES (crane picornavirus 5) (GenBank: KY312544.1) (genus *Grusopivirus*), (**C**) Model of the structure of the pemapivirus A1 IRES (Chinese softshell turtle picornavirus) (GenBank: MG600106.1) (genus *Pemapivirus*) and (**D**) Model of the structure of the symapivirus A1 (Wenling triplecross lizardfish picornavirus) (GenBank: MG600076.1) (genus *Symapivirus*), derived as described for related IRESs [114,130]. The initiation codon for the viral polyprotein is indicated by a rectangle. The nomenclature of domains and helices is as in [114]. Nucleotides are numbered, and sequence motifs in domain III of these IRESs that are conserved in other type IV IRESs, such as the conserved apical loops of subdomains IIIa, IIId, IIIe and the unpaired purine residues in helix III_1_ are indicated by gray shading, as is a loop-E motif in domain II of the grusopivirus A1 IRES. Base pairs that are conserved in the IRES of another pemapivirus, Chinese broad-headed pond turtle picornavirus (GenBank: MG600108.1) [141], due to compensatory nucleotide differences between it and Chinese softshell turtle picornavirus are indicated by light blue rectangles in panel C.

Diresapivirus A of the genus *Diresapivirus* was identified in bats [149], and its 5′UTR is similar (~60% nucleotide identity) to the 5′UTRs of bat sapelovirus (GenBank: KX644938.1) [144] and the sapelovirus-like bat picornavirus 1 (GenBank: HQ595340.1) [120]. The latter contains a slightly divergent type IV IRES [130], and the diresapivirus A IRES resembles it structurally (Figure 5A) in that domain II lacks a loop-E motif and domain III contains a conserved PK-IIId-IIIe-IIIf core but lacks the apical subdomain IIIc.

Grusopivirus A1 of the genus *Grusopivirus* was identified in red-crowned cranes [150]. It has a type IV IRES (Figure 5B) that consists of ~80nt-long domain II that contains a loop-E motif, and a divergent domain III that lacks subdomain IIIc and the apical ‘AGUA’ loop in subdomain IIIa, but contains all other typical structures and sequence motifs. Domains II and III are highly homologous (90% sequence identity) to analogous domains in the IRES of Lorikeet picornavirus 1 (GenBank: MK443503.1) [132], and in both viruses, are located immediately downstream of a stable, ~130 nt-long hairpin domain that is capped by a conserved ‘8-like’ motif ([132]; data not shown) that occurs at an analogous location in members of the genera *Aalivirus*, *Anativirus*, *Aquamavirus* and *Avihepatovirus* [129,130]. The PK-IIId-IIIe-IIIf core of the grusopivirus A1 IRES shares ~75% nucleotide identity with the corresponding region of type IV IRESs of members of the genera *Felipivirus* and *Sapelovirus* [121,130]. The adjacent coding sequence in grusopivirus A1 encodes the P1 capsid protein precursor, and it is most closely related to the P1 precursor encoded by members of the genus *Avihepatovirus* rather than, e.g., the genus *Sapelovirus*, hinting at an ancestral recombination event.

The PK-IIId-IIIe-IIIf core of the IRES of pemapivirus A1 (Chinese softshell turtle picornavirus) (genus *Pemapivirus*) [141] is also closely related to the equivalent core in IRESs from members of the genus *Sapelovirus* (~75% nucleotide identity) and it similarly lacks domain IIIc (Figure 5C). Unusually, domain II contains a small branching subdomain. The IRES is highly homologous (90% identity) to that of the pemapivirus Chinese broad-headed pond turtle picornavirus [139], and sequence covariation is extensive, so that IRES structure is maintained. The coding sequence adjacent to the IRES in pemapivirus A1 encodes an uncharacterized leader (L) protein followed by the P1 capsid protein precursor, which is most closely related to livupivirus and kobuvirus equivalents. Notably, the pemapivirus A1 leader and capsid proteins are not closely related to the (much shorter) sapelovirus L protein or to sapelovirus capsid proteins, respectively, suggesting that the structure of the Pemapivirus genome could reflect recombination near the IRES-ORF boundary.

The IRES in symapivirus A1 (Wenling triplecross lizardfish picornavirus) [141] of the genus *Symapivirus* resembles IRESs from members of the genera *Diresapivirus*, *Grusopivirus* and *Pemapivirus* in containing an extended domain II and a variant of domain III that lacks subdomain IIIc and the apical ‘AGUA’ loop in IIIa but has all other typical elements (Figure 5D). The PK-IIId-IIIe-IIIf core of this IRES shares 67% nucleotide identity with the IRES of anativirus A (GenBank: AY563023.1) [119] of the genus *Anativirus* (which is closely related to the genus *Sapelovirus*) whereas the capsid proteins are most closely related to those of the genus *Kobuvirus* (which is phylogenetically distant from the genera *Anativirus* and *Sapelovirus*).

Analysis of novel picornavirus sequences identified numerous even simpler, and therefore possibly primordial type IV IRESs. The type IV IRES of Guanxi changeable lizard picornavirus 2 (GCLPV2) (GenBank: MG600105) [141] (Figure 4B) exemplifies a group of short (~150 nt-long) type IV IRESs that contain domain II, the conserved PK-IIId-IIIe-IIIf core and a single sub-domain (IIIa) at the apex of domain III. Other members include Beihai conger picornavirus (GenBank: MG600065), Wenling lepidotrigla picornavirus (GenBank: MG600079), Wenling hoplichthys picornavirus (GenBank: MG600101) and Wuhan carp picornavirus (GenBank: MG600066), all isolated from fish [141]: there is 48% - 75% sequence identity from the 5′ border of the pseudoknot to the initiation codon in these four IRESs, and 41% - 49% identity in this region between them and the GCLPV2 (reptile) IRES. Although these IRESs superficially resemble the ~140 nt-long limnipivirus IRESs, in which the apical region of domain is also truncated [129], sequence identity between members of the two groups is low (33%-45%). However, the segment of the GCLPV2 IRES from domain IIId to the initiation codon shares 70%–75% nucleotide sequence identity with analogous regions of IRES from members of the genus *Felipvirus* (e.g., feline picornavirus 1 (GenBank NC_016156)) [121], from confirmed and potential members of the genus *Sapelovirus* (e.g., sapelovirus B (GenBank: LC503602) [151] and Ia io picornavirus 1 (GenBank: JQ814852)) [152] as well as from confirmed and possible members of the genus *Pemapivirus*. The GCLPV2 IRES shares more extensive homology, including all of domain III, with IRESs from several avian picornaviruses, although the level of nucleotide identity (52%–59%) is lower. They include the unclassified Pink-eared duck picornavirus (GenBank: MK204421) [148] (Figure 4C) and Avocet picornavirus (GenBank: MH453807) [153], anativirus A (GenBank: NC_006553) [115] and the sapelovirus-like phacovirus (GenBank: KT880670) [154]. The leader protein encoded immediately downstream of the GCLPV2 IRES is shorter and/or lacks homology to equivalents in the other avian picornaviruses, whereas there is strong homology in the downstream capsid protein region.

Taken together, it is evident that members of numerous picornavirus genera contain sapelovirus-like type IV IRESs, in which the PK-IIId-IIIe-IIIf core is strongly conserved and the apical region consists of one or two stemloop subdomains rather than the four-way ‘IIIabc’ junction that is characteristic of HCV-like IRESs.

## 9. Transfer of Heterologous IRESs between the *Pegivirus* and other Genera of *Flaviviridae*

The *Pegivirus* genus of *Flaviviridae* contains 11 species, designated *Pegivirus A*–*K*, and numerous unclassified members [155]. They infect mammals, and although they commonly cause persistent infections, generally have little or no associated pathogenesis [156]. Pegiviruses have a positive-strand RNA genome with a structure that closely resembles that of hepaciviruses: long untranslated regions flank an open reading frame encoding a single, large polyprotein that is proteolytically processed to yield mature structural and non-structural proteins. The order of genes in hepacivirus and pegivirus genomes is the same, except that pegiviruses do not encode an N-terminal Core protein (although pegivirus F, pegivirus G and pegivirus H encode basic proteins at the equivalent N-terminal location in the polyprotein), and some pegiviruses may encode an additional structural protein (‘X’) between structural and non-structural proteins [157,158,159].

**Figure 6:**
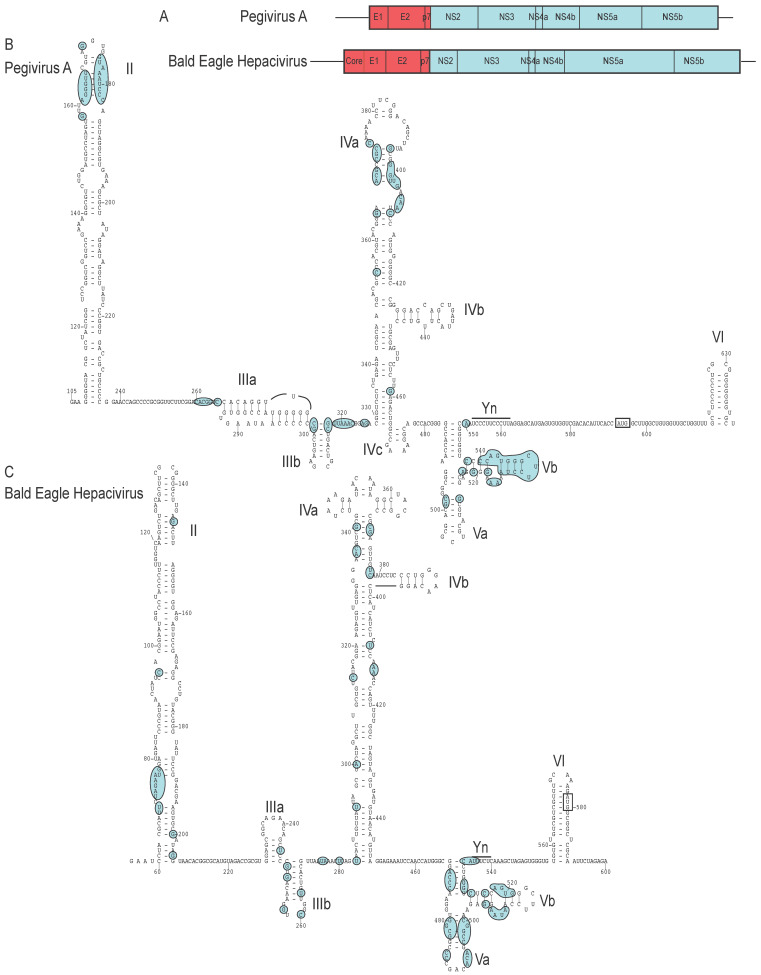
Structurally related IRESs in Pegivirus A and Bald eagle hepacivirus (**A**) Schematic representations of pegivirus A and Bald eagle hepacivirus (BeHCV), showing the ORFs encoding the polyprotein in each, the individual proteins that are cleaved from each by cellular and viral proteins, the flanking noncoding regions flanking the ORFs. Note that the IRESs are located adjacent to unrelated coding sequences, namely the pegivirus E1 envelope glycoprotein and the BeHCV Core protein, respectively. (**B**) Secondary structure model of a segment of the 5′UTR of pegivirus A [83] (NCBI ref. seq. NC_001837.1) and (**C**) secondary structure model of a segment of the 5′UTR of BeHCV (GenBank: MN062427). A similar model to that shown for BeHCV has been proposed for the 5′UTR of Duck hepacivirus [160]. Domains are labeled II – VI, and nucleotides are numbered at 20 nt. intervals. The initiation codon is boxed and the pyrimidine tract is labeled Yn. The shaded elements indicate nucleotides that are conserved in (B) six or more of pegivirus A (NCBI ref. seq. NC_001837.1), pegivirus B (GenBank: KC796089.1), pegivirus C (GenBank: HM638236.1), pegivirus D (NCBI ref. seq.: NC_038433), pegvirus I (NCBI ref. seq.: NC_038437.1), pegivirus K (NCBI Ref. seq. NC_034442.1) and Dolphin pegivirus isolate DPgV (GenBank: MK059751.1)and (C) in seven or more of Bald eagle hepacivirus isolate NA03-001 (GenBank: MN062427.1), Duck hepacivirus strain HCL-3 (GenBank: MK737641.1), hepacivirus F (NCBI Ref. seq. NC_038427.1), hepacivirus J isolate hepacivirus/RMU10-3382/GER/2010 (NCBI Ref. seq. NC_038429.1), hepacivirus sp. (from *Trachemys scripta elegans*) (GenBank: MG334001), Jogalong virus isolate P1-1 (GenBank: MN133813.1), Rodent hepacivirus isolate hepacivirus/NLR08-365/NEL/2008 (GenBank: KC411796) and *Sigmodontinae* hepacivirus strain On/2012 (GenBank: MH370348.1).

The structurally related 5′UTRs of pegivirus A and C both contain IRESs [83,161]. 5′UTRs with a similar domain organization occur in pegivirus D [162], pegivirus E [163], pegivirus I [164], pegivirus K [163], numerous isolates of Pegivirus B [164], as well as in the 5′UTRs of currently unclassified pegiviruses, for example from pigs and dolphins [165,166]. These 5′UTRs contain two extended hairpin domains (II and IV) flanking a pair of smaller stemloops (IIIa and IIIb), the first of which may form part of a pseudoknot (Figure 6A). The conserved Y-shaped domain Va/Vb is commonly, but not invariably, followed by a pyrimidine tract that forms part of a Yn-Xm-AUG motif. In some pegivirus genomes, the initiation codon for the viral polyprotein is followed by a hairpin domain VI that could potentially enhance translation by preventing further 5′-3′ scanning by ribosomal initiation complexes. Although the combination of a Y-shaped domain and the Yn-Xm-AUG motif resembles the 3′-terminal region of type II IRESs, there is no evidence that pegivirus domain V binds analogously to eIF4G/eIF4A or that the pyrimidine tract acts the landing site for ribosomes. In addition to this structural conservation, several sequence motifs in these pegivirus IRESs are highly conserved, including motifs in domains IIIa/IIIb, at the apex of domain IVa and in domain Vb (Figure 6A). The factor interactions and mechanism of action of this class of pegivirus has not been characterized, and the function of conserved motifs and structures remains unknown.

Several hepaciviruses also contain pegivirus-like IRESs, as first noted for hepacivirus F, hepacivirus J and other rodent-infecting hepaciviruses [164], for human hepacivirus H [159] and for three Duck hepacivirus strains [160]. We identified additional pegivirus IRES-like sequences in Bald Eagle hepacivirus (BeHV) [167] (Figure 6B), in the putatively avian Jogalong virus [168] and in hepaciviruses from *Oliogoryzomys nigripes* (black-footed pygmy rice rat) [169] and *Trachemys scripta elegans* (red-eared terrapin) (GenBank: MG334001). The predicted structure of these elements resembles that of the canonical pegivirus A 5′UTR (Figure 6A), although domains IIIa/IIIb and the apex of domain IVa are truncated in some of the hepacivirus 5′UTRs. However, domain V and the adjacent Yn-Xm-AUG motif are strongly conserved (except in BeHV, which has a short Yn motif and a GUG triplet in place of the AUG triplet), and the most strongly conserved nucleotides in these putative hepacivirus IRESs are located at analogous positions to those in the pegivirus IRESs (Figure 6A,B), suggesting that they have the same functional role. The coding sequences adjacent to these hepacivirus 5‘UTRs encode the Core protein, and their N-terminal regions are non-conserved and variable in length, suggesting that the recombination breakpoint lies in this area of the genome, and that hepaciviruses may have acquired pegivirus-type IRESs by horizontal gene transfer on multiple occasions.

**Figure 7:**
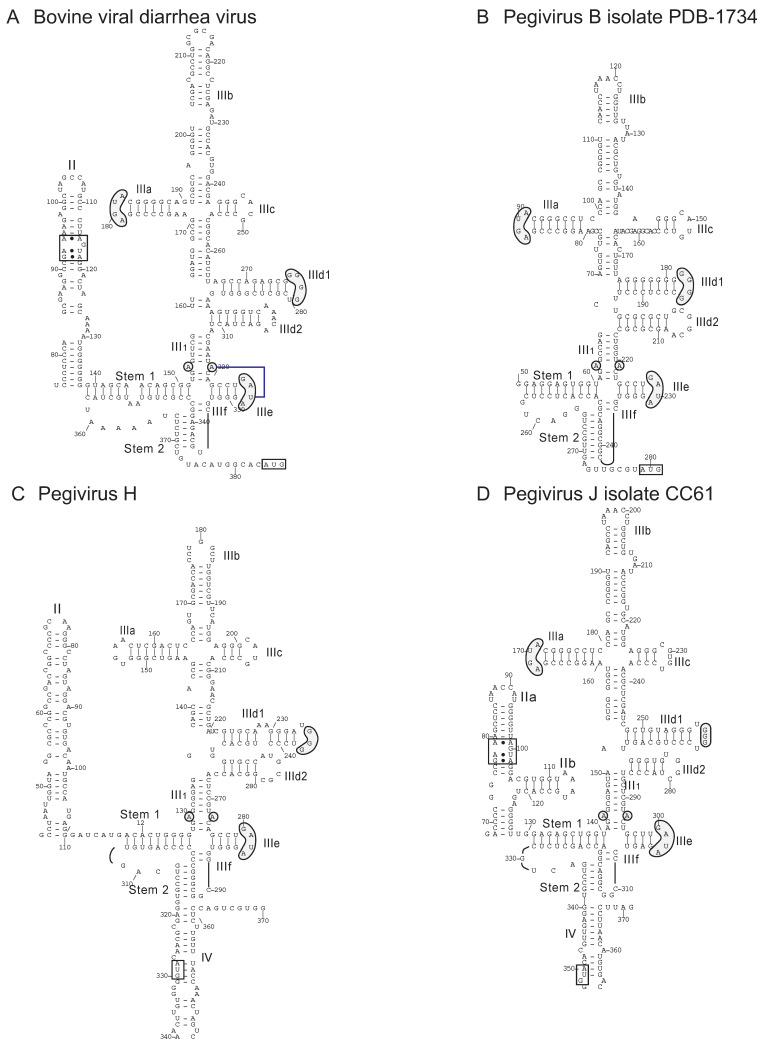
Models of the type IV IRESs of bovine viral diarrhea virus of the genus *Pestivirus* and related type IV IRESs in members of the genus *Pegivirus* of *Flaviviridae*. The models show the structures of the IRESs of (**A**) bovine viral diarrhea virus (BVDV isolate Carlito) (GenBank: KP313732.1), (**B**) pegivirus B isolate PDB-1734 (GenBank: KC796087.1), (**C**) pegivirus H (human pegivirus 2) (GenBank: KT427409.1) and (**D**) pegivirus J isolate CC61 (GenBank: KC815311.1), in the last instance as previously proposed [170]. The nomenclature of domains and helices is as in [114]; nucleotides are numbered at intervals and the initiation codon is boxed. The loop-E motif in domain II [58] is labeled and conserved sequence motifs in domain III, such as the apical loops in subdomains IIIa, IIId and IIIe, and the unpaired purine residues in helix III_1_ are indicated by grey shading. The tertiary base-pairing interaction between the unpaired purine residue in helix III_1_ and loop IIIe is indicated by blue line. The pegivirus B sequence is incomplete, so that a model of domain I could not be proposed.

As a counterpoint to these observations, it is apparent that as well as acting as IRES donors to other members of *Flaviviridae*, pegiviruses may also have acquired IRESs from members of this family. Thus, a type IV IRES was identified in the pegivirus J genome [170] and our analysis revealed that several other pegivirus genomes also contain type IV IRESs, including pegivirus F, pegivirus H, a member of *Pegivirus B* (GenBank: KC796087.1) and some currently unclassified rodent pegiviruses [157,158,169,171]. Sequence identity ranges from ~50%–65%, and although some sequences are incomplete, it is evident that they all adopt similar structures (Figure 7), including a pestivirus-like domain IIId2. Domain II is least conserved, and in some instances, the initiation codon is sequestered in domain IV. The loop-E motif and the apical ‘AGUA’ motif in subdomain IIIa is absent in some instances, but these IRESs contain all of the other hallmarks of canonical type IV IRESs. The strongest homology is with the IRES of BVDV, a member of the *Pestivirus* genus of *Flaviviridae* (~62% nucleotide identity with Pegivirus B and Pegivirus J).

The presence of two types of IRES in the genomes of members of the genus *Pegivirus* that also occur in members of the genus *Hepacivirus* suggests that pegiviruses can act as donors and as recipients of IRESs to and from other viral genomes.

## 10. Conclusions

Molecular epidemiological studies established that the junction of the 5′UTR and the open reading frame that encodes the viral polyprotein constitutes a hot spot for recombination in picornaviruses. Intratypic recombination leading to acquisition of novel 5′UTRs by these viruses has been extensively documented, establishing the important concept that the 5′UTR is a distinct genetic unit that evolves independently and at a different rate to other elements of the viral genome (e.g., [84]). Intertypic recombination occurs less efficiently and is consequently less frequently observed but has been associated with the appearance of novel viral species. These recombination events involved transfer of IRESs of the same class within the same genus. However, the observation that a member of the genus *Teschovirus* of *Picornaviridae* has a type IV IRES [110], which had until then only been identified in members of the genera *Hepacivirus* and *Pestivirus* of *Flaviviridae* suggested that recombinational transfer of IRESs could even occur between different virus families. Subsequent analyses have revealed type IV IRESs in many other genera of *Picornaviridae*, and with the identification here of type IV IRESs in a further seven genera (*Crohivirus*, *Diresapivirus*, *Grusopivirus*, *Mosavirus*, *Pemapivirus*, *Symapivirus* and *Tropivirus*), it is now evident that they are very widely distributed in this family. The presence of structurally distinct IRESs appended to homologous coding sequences and or related IRESs linked to different coding sequences in viral genomes strongly suggests that IRESs have moved between them by horizontal gene transfer. Significantly, several genera of *Picornaviridae* have one or more members that contain a type IV IRES in addition to other members that contain an IRES of either type I, II or III. The complementary observations [159,161,163,164] summarized and extended here that some members of the genus *Pegivirus* contain pestivirus-like type IV IRESs (Figure 7) whereas some members of the genus *Hepacivirus* contain pegivirus-like IRESs (Figure 6) strongly support the conclusion that IRESs are independent genetic entities that can move between viral genomes by horizontal gene transfer. The presence of type IV IRESs in multiple genera of *Picornaviridae* as well as in three genera of *Flaviviridae* is of particular interest.

The structures of the type IV IRESs reported previously and identified here in additional genera of *Picornaviridae* and *Flaviviridae* confirm previous conclusions made concerning their conserved and variable elements. The most conserved region comprises the pseudoknot and subdomains IIId, IIIe and IIIf, which are critical for binding of the IRES to the 40S subunit and for positioning the initiation codon in the P site and the proximal coding region in the mRNA-binding cleft of the 40S subunit. The pegivirus type IV IRESs have most or all of the key motifs present in canonical type IV IRESs (Figure 7), whereas picornavirus type IV IRESs are more divergent. Thus, the apical GGG motif in subdomain III that base pairs with ES7 of 18S rRNA is reduced to a GG dinucleotide in members of *Pemapivirus* (Figure 5C) and the GA[U/C]A loop at the apex of subdomain IIIe is not conserved in members of the genera *Aalivirus* and *Megrivirus* [130]. Nevertheless, these elements are all still capable of engaging in critical interactions with 18S rRNA and within the IRES, respectively. By contrast, there is significant structural heterogeneity in elements that have accessory rather than essential functions, such as domain II and subdomain IIId2 (e.g., [134,137,172,173]), and several picornavirus type IV IRESs lack one or two of the subdomains that conventionally radiate from the IIIabc four-way junction. Members of the genera *Aalivirus*, *Aquamavirus*, *Avihepatovirus* and *Grusapivirus* contain a highly conserved ‘8-like’ motif at the apex of a long hairpin immediately upstream of the IRES (e.g., [130,132]) that in avihepatovirus augments IRES function [138]. How it functions, and how initiation complexes assemble on IRESs that lack binding determinants for eIF3 and the 40S subunit in apical regions of domain III, remain to be determined.

A key conclusion arising from the observations reported here and elsewhere (e.g., [130]) is that IRESs appear to have undergone recombinational exchange between viral genomes on multiple occasions. Although experimental studies of the effects of IRES exchanges on viral phenotype have revealed that they can impair viral replication and lead to restricted activity in some cell types, so that a heterologous IRES may not be beneficial immediately after acquisition, additional adaptive mutations in the IRES or other elements of the genome may be necessary to optimize activity in the new genetic and physical environment. Such changes could include the appearance of synonymous substitutions to optimize the proximal coding region for IRES function (e.g., [174]), regeneration of a disrupted Yn-Xm-AUG motif or, in the case of IRESs that depend on specific interactions with initiation factors, substitutions that optimize interactions with the likely somewhat divergent proteins from the new host, or substitutions that mimic ITAF-induced conformational changes.

Although the direction of movement of IRESs between viral genomes has not definitively been established, the widespread distribution of type IV IRESs in two different virus families and in multiple genera of *Picornaviridae*, and their presence in genera that predominantly utilize a different type of IRES together suggest that type IV IRESs have repeatedly been acquired by members of *Picornaviridae* and possibly, by members of the genus *Pegivirus* of *Flaviviridae*. Several factors could favor acquisition of type IV IRESs. Recombination that involves a replicative mechanism would be limited to viruses that can infect the same cell. In this respect it may be important that type IV IRESs depend on interaction with a widely conserved element in ES7 of 18S rRNA: this may account for the widespread distribution of this class of IRES in viruses that infect amphibians, birds, fish, reptiles and mammals. Exploitation of this type of interaction would lead to fewer host cell restrictions on function than those encountered by IRESs that depend primarily on interactions with initiation factors, and particularly, with ITAFs that may be expressed in a cell type-specific manner. Utilization of a type IV IRESs may therefore be beneficial by minimizing host cell restrictions on translation of viral proteins. Type IV IRESs are also significantly more active than type I and type III IRESs, and moreover, are notably resistant to inhibition by phosphorylation of eIF2 [65,72], a common virus-induced innate immune response.

As noted previously [130], recombination could also lead to the exchange or de novo acquisition of IRES domains or subdomains. The ability of the HCV IRES to function after substitution of domain II by the analogous domain from other type IV IRESs [175,176] indicates that this is feasible. Moreover, we have noted above that domain II is much more variable than domain III, and that homology between IRESs from different viral species is commonly restricted to the PK-IIId-IIIe-IIIf core. Such a scenario, which could involve a combination of non-replicative and replicative recombination process, could account for the capture and dissemination of the extended and highly conserved ‘8-like’ motif that occurs at the apex of domain IIIb in members of the genus *Megrivirus* but at the apex of a hairpin upstream of domain II in members of the genera *Aalivirus, Anativirus*, *Aquamavirus* and *Avihepatovirus* [129,130,132].
